# Simultaneous Bilateral Proximal Humerus Fractures Treated with Single-Stage Bilateral Reverse Shoulder Arthroplasty

**DOI:** 10.1155/2022/2162331

**Published:** 2022-04-20

**Authors:** Taiki Tokuhiro, Atsushi Urita, Yusuke Kameda, Makoto Motomiya, Naoya Watanabe, Norimasa Iwasaki

**Affiliations:** ^1^Department of Orthopedic Surgery, Faculty of Medicine and Graduate School of Medicine, Hokkaido University, Sapporo, Japan; ^2^Department of Orthopedic Surgery, Dokkyo Medical University, Mibu, Japan; ^3^Department of Orthopedic Surgery, Obihiro Kosei Hospital, Obihiro, Japan

## Abstract

**Introduction:**

Simultaneous bilateral fractures of the proximal humerus are infrequent, and simultaneous bilateral three- or four-part fractures are even rarer. Reverse shoulder arthroplasty (RSA) is being used increasingly for the treatment of three- and four-part fractures of the proximal humerus. However, treatment of simultaneous bilateral fractures of the proximal humerus is difficult because of concern about postoperative immobilization and rehabilitation. *Case Presentation*. A 75-year-old woman presented with bilateral shoulder pain subsequent to a fall on the street. Physical examination and radiographs showed simultaneous bilateral fractures of the proximal humerus. The right side fracture was classified as a four-part fracture and the left side fracture as a three-part fracture, according to Neer's classification. The right shoulder had a risk of avascular necrosis of the humeral head. For the left shoulder, the fracture type had caused ischemia of the humeral head. Single-stage bilateral RSA was performed 9 days after the injury. An abduction pillow was applied for 5 weeks postoperatively. Passive motion exercises were permitted starting at 4 weeks postoperatively, and active range of motion exercises were permitted at 6 weeks postoperatively. At the patient's most recent follow-up 30 months after surgery, the patient reported no restriction of the activities of daily living. Radiographs revealed no lucent line on the humerus and glenoid components, although bone resorption and superior retraction of the tuberosities on both sides were observed.

**Conclusions:**

Single-stage bilateral RSA improved shoulder function, but healing of the greater tuberosity can affect the improvement in external rotation after the operation. Although a long-term follow-up is needed, single-stage bilateral RSA appears to be a viable treatment option.

## 1. Introduction

Proximal humerus fractures are among the most common fractures in elderly patients with an incidence of 82 per 100,000 persons per year [1]. Most of these fractures are categorized as one- or two-part fractures according to Neer's classification [2], whereas four-part fractures are uncommon and comprise around 3% of all proximal humerus fractures [3, 4]. Simultaneous bilateral fractures of the proximal humerus are infrequent, and simultaneous bilateral three- or four-part fractures are even rarer, and only a very few reports have been published [5–7]. Surgical treatment, such as osteosynthesis or shoulder arthroplasty, is often needed to treat complex three- and four-part fractures of the proximal humerus [8, 9]. Humeral head replacement (HHR) is performed in cases involving the risk of avascular necrosis of the humeral head; however, tuberosity malposition, which can occur in HHR, leads to poor clinical outcomes [10].

Treatment of simultaneous bilateral proximal humeral fractures is difficult because bilateral shoulders are required to be immobilized after surgery. Limited postoperative treatment after bilateral surgery could lead to inferior improvement in shoulder function compared with that for a unilateral injury. However, only a few case reports of simultaneous proximal humerus fractures treated with bilateral RSA have been published [11–13]. Here, we report a case of simultaneous bilateral three- and four-part fractures of the proximal humerus that were treated with single-stage bilateral RSA. The patient was informed that data concerning her case would be submitted for publication, and she provided consent.

## 2. Case Presentation

A 75-year-old right-handed dominant woman was referred to our emergency department from a primary care hospital with the diagnosis of bilateral proximal humerus fractures subsequent to a fall on the street. She had slipped and landed on her outstretched arms but experienced no direct trauma in either shoulder. The patient had a history of hypertension, cholelithiasis, and left femoral neck fracture, with no previous shoulder pathology. She had no restrictions of her activities of daily living before the trauma. Clinical examination showed bilateral severe shoulder pain and restricted shoulder motion but no neurovascular deficits including that of the axillary nerve.

**Figure 1:**
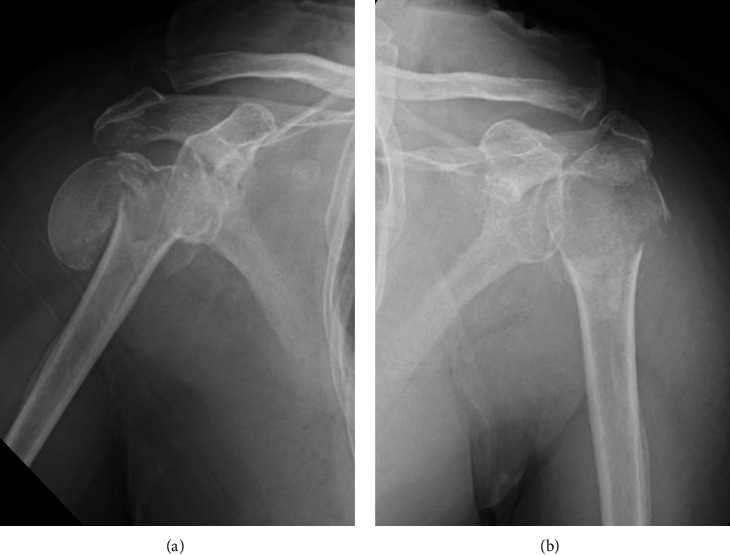
Radiographs showing bilateral proximal humeral fractures: (a) right shoulder and (b) left shoulder.

**Figure 2:**
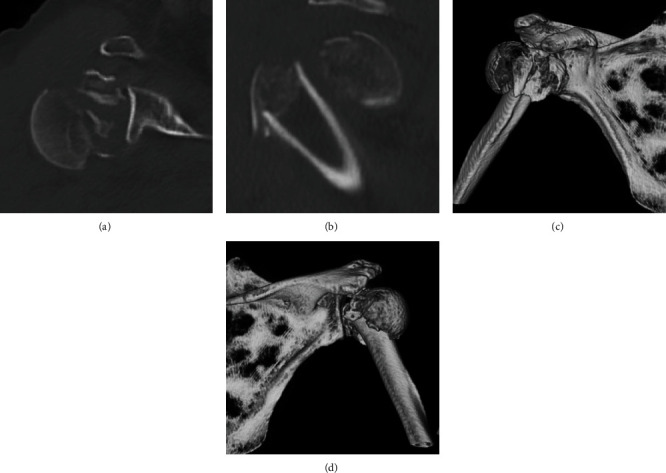
Computed tomography images of the right shoulder showing the four-part fracture with dislocation of the proximal humerus: (a) axial image, (b) sagittal image, (c) frontal view in a three-dimensional (3D) image, and (d) posterior view in a 3D image.

**Figure 3:**
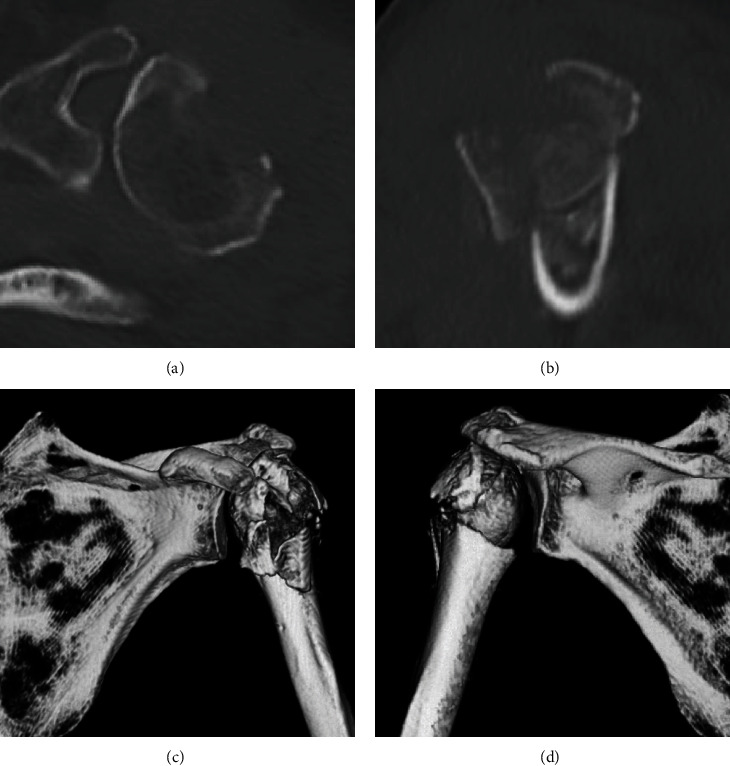
Computed tomography images of the left shoulder showing the three-part fracture of the proximal humerus: (a) axial image, (b) sagittal image, (c) frontal view in a 3D image, and (d) posterior view in a 3D image.

Radiographs showed bilaterally displaced proximal humerus fractures with dislocation of the right humeral head (Figures 1(a) and 1(b)). Computed tomography revealed a fracture dislocation with displaced comminuted fracture of the greater and lesser tuberosities in the right shoulder (Figures 2(a)–2(d)) and varus displacement of the humeral head from disruption of the medial calcar support and displacement of the greater tuberosity in the left shoulder (Figures 3(a)–3(d)). According to Neer's classification [2], the right side fracture was classified as a four-part fracture and the left side fracture as a three-part fracture. There were no obvious degenerative changes in either glenohumeral joint on radiographs. The limbs were immobilized in arm slings, and the patient was admitted to our hospital to treat the bilateral proximal humerus fractures.

The treatment options were discussed in the context of the patient's age and types of fractures. We considered that surgical treatment was appropriate for these bilateral fractures. According to Hertel's criteria [14], the right shoulder had a risk of avascular necrosis (AVN) of the humeral head because of the dislocation of the humeral head without medial calcar support and the comminuted fracture of the greater and lesser tuberosities. The fracture pattern and patient's age suggested the need for prosthetic replacement rather than osteosynthesis. Because HHR may not necessarily improve shoulder function, we considered that an RSA would improve the clinical results and provide better shoulder function than HHR, and we decided to use RSA on the right side. For the left shoulder, conservative treatment might be an option; however, the fracture type had caused ischemia of the humeral head because of disruption of the medial calcar support. In our hospital, head-preserving surgery (osteosynthesis) is indicated mainly when there is sufficient bone quality for performing a stable osteosynthesis. Therefore, we planned to perform the osteosynthesis but to use an RSA if the bone quality was found to be insufficient.

**Figure 4:**
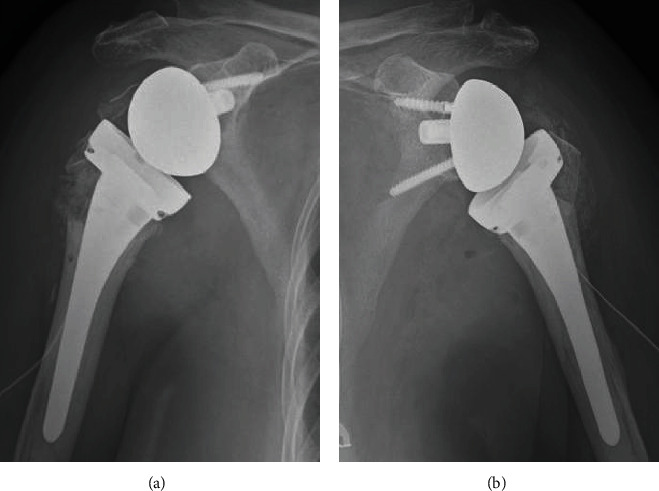
Postoperative radiographs after bilateral reverse shoulder arthroplasty: (a) right shoulder and (b) left shoulder.

She was operated on 9 days after the injury. The operation started on the right side. With the patient under general anesthesia, a deltopectoral approach was performed with the patient in the beach-chair position. The RSA was undertaken using the Medacta Shoulder System (Medacta International, Castel San Pietro, Switzerland). After the baseplate and glenosphere were placed, the cemented humeral stem was implanted. The tuberosities were then sutured to the humeral shaft using suture tapes (Arthrex, Naples, FL, USA) and through the dedicated holes on the stem. The greater and lesser tuberosities were sutured to the prosthetic stem using four No. 2 nonabsorbable sutures. Cancellous chips from the humeral head were packed between the humeral shaft and the tuberosities (Figure 4(a)).

The operation on the left side was performed next. Using a deltopectoral approach, we evaluated the fracture pattern and bone quality. The humeral head was found to be unstable, the medial hinge was disrupted, the greater tuberosity was comminuted, and the bone quality was poor. Therefore, we considered a cessation of blood supply to the humeral head and decided to use an RSA for the left side using the same prosthesis as for the right side (Figure 4(b)). The intra- and postoperative blood loss was 550 mL, and the patient required no blood transfusion.

**Figure 5:**
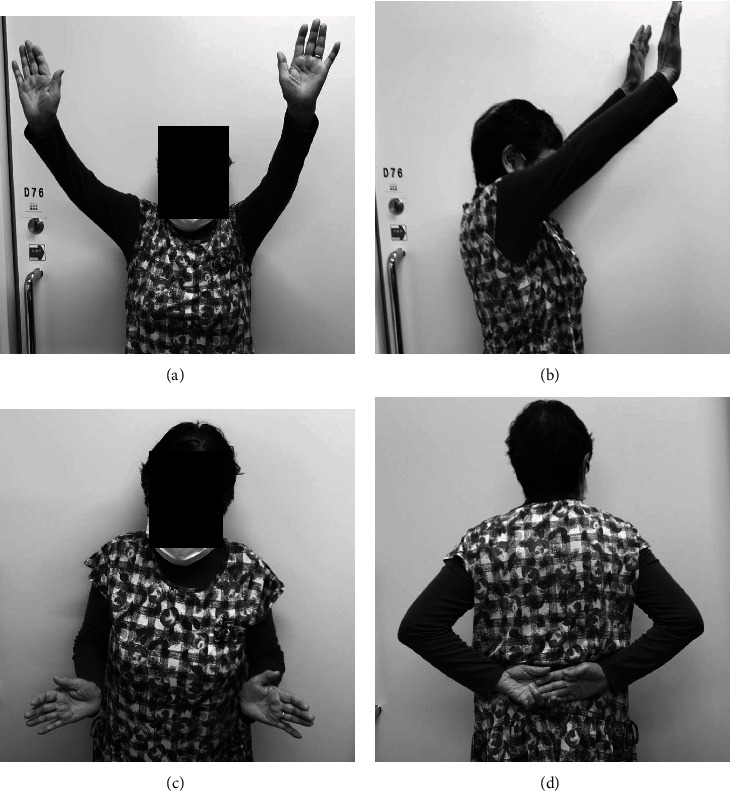
Active shoulder range of motion at the most recent follow-up: (a) forward elevation, (b) abduction, (c) adducted external rotation, and (d) adducted internal rotation.

**Figure 6:**
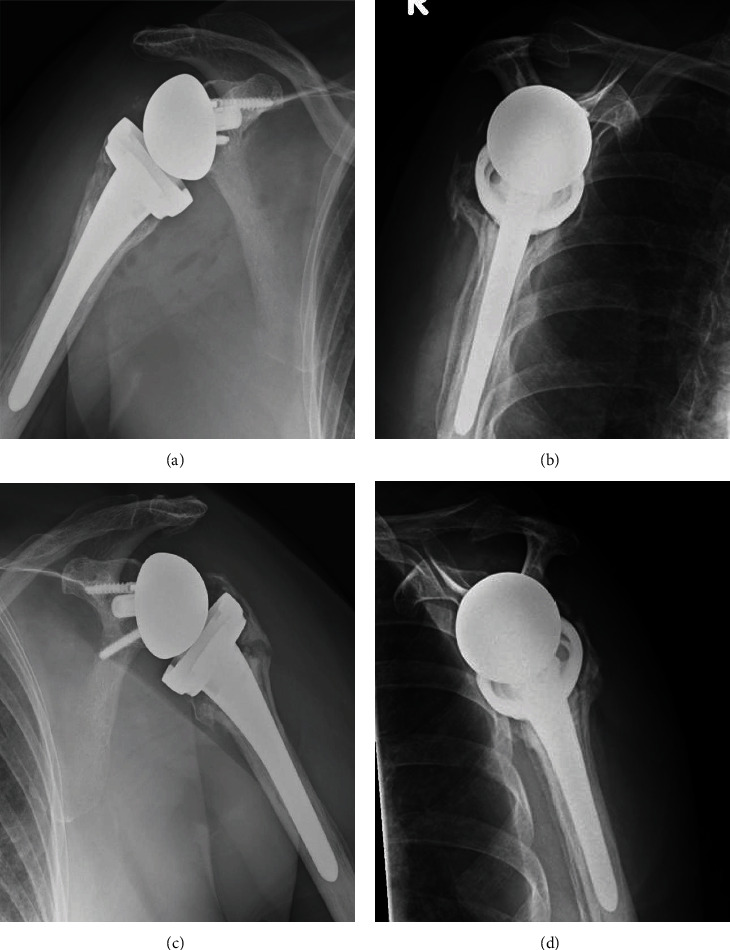
Radiographs showing both shoulders at the most recent follow-up: (a) Grashey view and (b) transscapular Y view of the right shoulder and (c) Grashey view and (d) transscapular Y view of the left shoulder.

An abduction pillow was applied for 5 weeks postoperatively. Passive motion exercises were permitted starting at 4 weeks postoperatively, and active range of motion exercises were permitted at 6 weeks postoperatively. At the patient's most recent follow-up 30 months after surgery, she reported no pain in either shoulder. The active range of motion was 110 degrees of flexion, 110 degrees of abduction, -10 degrees of external rotation, and L4 for internal rotation on the right side and 120 degrees of flexion, 120 degrees of abduction, -10 degrees of external rotation, and L3 for internal rotation on the left side (Figures 5(a)–5(d)). The patient reported no restriction of the activities of daily living. Radiographs revealed no lucent line on the humerus and glenoid components, although bone resorption and superior retraction of the tuberosities on both sides were observed at the final follow-up (Figures 6(a)–6(d)).

## 3. Discussion and Conclusions

Simultaneous bilateral proximal humerus fractures are rare and account for only 1% of all humerus fractures. Proximal humerus fractures generally occur as a result of high-energy injury in young adults and low-to-moderate energy events combined with low bone quality in the older people. Simultaneous bilateral proximal humerus fractures have been reported to be associated with a dislocation secondary to a seizure episode, electrocution, and extreme trauma, called the “triple E syndrome” (epilepsy, electrocution, and extreme trauma) [6, 15, 16]. This patient's injuries did not involve this syndrome, but her simultaneous bilateral humerus fractures were caused by a ground-level fall coupled with her osteoporosis.

Osteosynthesis using a locking plate or intramedullary nailing is usually considered when treating three- and four-part fractures. However, the disruption of blood supply in the humeral head can lead to AVN of the humeral head, which is a well-known complication of comminuted and displaced proximal humerus fractures. Hertel et al. reported that the most relevant predictors of ischemia after osteosynthesis are the calcar length, medial hinge integrity, and some specific fracture types [14]. In this case, the bilateral humeral head fractures had increased the risk of AVN of the humeral head after osteosynthesis.

Regarding shoulder arthroplasty for the treatment of three- and four-part fractures, HHR has been used in the past [8, 9]; however, tuberosity malposition and migration lead to poor clinical outcomes [10]. By contrast, RSA for proximal humerus fractures provides superior forward elevation and has lower complication rates compared with HHR [17–20]. Therefore, RSA is being used increasingly to treat complex proximal humerus fractures [21–25].

In the rare situation of simultaneous bilateral proximal humerus fractures, the choice of treatment is more controversial but should be performed as for a unilateral fracture. A few case reports have reported on the conservative treatment, closed reduction and percutaneous fixation, open reduction and internal fixation, osteochondral autograft, and HHR for bilateral fractures [5–7, 26–28]. Cases of bilateral RSA for the treatment of simultaneous bilateral proximal humeral fractures have also been reported [11–13]. These reports have described that bilateral RSA improves shoulder function over the short term. Similarly, our patient recovered shoulder function and reported on no restriction of the activities of daily living; however, the improvement in external rotation was poor because of bone reabsorption and partial retraction of the tuberosities after surgery.

In bilateral shoulder arthroplasty, the timing of the second arthroplasty can affect the clinical outcomes. Walters et al. reported that a second arthroplasty within 3 months leads to higher complication rates such as the need for revision surgery because of loosening/lysis, periprosthetic fracture, venous thromboembolic events, and blood transfusions [29]. By contrast, Gerber et al. found that single-stage bilateral shoulder arthroplasty improved outcomes but had a higher rate of the need for transfusions compared with staged arthroplasty [30]. In the rare situation of simultaneous bilateral proximal humerus fractures, a longer waiting time should be avoided because of the risk of bone resorption of the greater tuberosity after surgery.

The healing of the tuberosities after RSA affects the postoperative range of motion, especially forward elevation and external rotation [31, 32]. The migration and subluxation of the tuberosities were more frequent in the early mobilization [33]. Immobilization of the shoulder joint would be needed for the healing of tuberosities in patients with severe osteoporosis. However, immobilization of both shoulder joints is difficult because of the need to maintain the activities of daily living, and prolonged immobilization may cause bone resorption in or retraction of the greater and lesser tuberosities. In this patient, poor bone quality in the tuberosities and the insufficient immobilization of both shoulders caused bone reabsorption in and retraction of the tuberosities after surgery. As a result, the improvement of external rotation was poor on both sides. Despite this limitation, this patient was satisfied with the treatment. However, postoperative treatment might have to be altered from that used for unilateral fractures.

In conclusion, we have described our experience treating a patient with simultaneous bilateral proximal humerus fractures. Single-stage bilateral RSA improved shoulder function, but healing of the greater tuberosity can affect the improvement in external rotation after the operation. Although a long-term follow-up is needed, single-stage bilateral RSA appears to be a viable treatment option.

## Data Availability

All data concerning the case are presented in the manuscript.
